# The Clinical Significance of Urinary Retinol-Binding Protein 4: A Review

**DOI:** 10.3390/ijerph19169878

**Published:** 2022-08-11

**Authors:** Krzysztof Ratajczyk, Andrzej Konieczny, Adrian Czekaj, Paweł Piotrów, Marek Fiutowski, Kornelia Krakowska, Paweł Kowal, Wojciech Witkiewicz, Karolina Marek-Bukowiec

**Affiliations:** 1 Department of Urology, Regional Specialist Hospital in Wroclaw, Kamienskiego 73a, 51-124 Wroclaw, Poland; 2 Department of Nephrology and Transplantation Medicine, Wroclaw Medical University, Borowska 213, 50-556 Wroclaw, Poland; 3 Research and Development Center, Regional Specialist Hospital in Wroclaw, Kamienskiego 73a, 51-124 Wroclaw, Poland

**Keywords:** urinary RBP4, renal diseases, biomarker

## Abstract

Effective biomarkers for early diagnosis, prognostication, and monitoring in renal diseases (in general) comprise an unmet need. Urinary retinol-binding protein 4, which is the most sensitive indicator of renal tubular damage, holds great promise as a universal biomarker for renal pathologies, in which tubular injury is the driving force. Here, we summarize the most important existing data on the associations between urinary retinol-binding protein 4 and renal diseases and highlight the untapped potential of retinol-binding protein 4 in clinical use.

## 1. Introduction

Retinol-binding protein 4 (RBP4) is a small (~21 kDa) plasma protein, first identified and described by Masamitsu Kanai in 1968 [1]. RBP4 belongs to lipocalins, a superfamily of protein transporters with an affinity for specific small lipophilic compounds such as retinoids, lipids, steroids, and bilins [2]. The protein is produced mainly in the liver (hepatokine) and, to a lesser extent, synthesized by adipocytes (20–40%, adipokine) and immune cells, i.e., macrophages [2,3,4]. RBP4 is a well-characterized principal transporter of all-trans-retinol (vitamin A alcohol, ROH), which also possesses fatty acid transport activity [1,5,6]. In normal kidneys, the concentration of RBP4 in plasma is relatively stable, high, and equals approximately 3–4 mg per 100 mL of plasma [1]. In circulation, under physiological conditions, a prevalent fraction of RBP4 (~86% molecules) remains saturated with retinol (holo-RBP4) and firmly bound to transthyretin (TTR, prealbumin, carrier of thyroid hormone), forming a 76 kDa complex. The large size of the “retinol transport unit” prevents renal clearance of RBP4 and ensures maintaining adequate retinol levels in the plasma [7]. Conversely, the retinol-free fraction of RBP (apo-RBP4) is subjected to glomerular filtration, efficient reabsorption, and degradation in the renal proximal tubules [8,9,10]. Only trace amounts of plasma RBP4 (~0.1 mg/24 h, 0.025%) escape the reabsorption/degradation route and undergo excretion in the urine [11]. The “runway” fraction of RBP4 (urinary RBP4, uRBP4) has particular importance from a clinical point of view, as its elevation in urine indicates tubular injury and reflects the severity of proximal tubular dysfunction [12]. A significant number of studies evidenced that uRBP4 comprises the most sensitive functional marker of renal tubules. Urinary RBP4 levels were found to be significantly increased (even >10^4^-fold) in a plethora of human diseases, affecting kidneys in both a direct or an indirect manner, e.g., glomerulopathies, prediabetes, diabetes, renal allograft dysfunction, chronic kidney disease, preeclampsia, renal cancer, and many others [13,14,15].

This review combines the representative up-to-date knowledge regarding uRBP4 concentration changes in human health and diseases and draws conclusions about the possible future use of uRBP4 as a nonspecific tool to evaluate human health. The detailed information regarding the biological functions of RBP4, its role in human health, as well as methods used for uRBP4 quantitation (and their shortcomings), has been recently summarized elsewhere and will not be discussed here [14,16,17].

Our aim was to provide an overview of the state of knowledge regarding uRBP4 in human health and diseases and to highlight its potential for clinical use.

## 2. Materials and Methods

**Figure 1:**
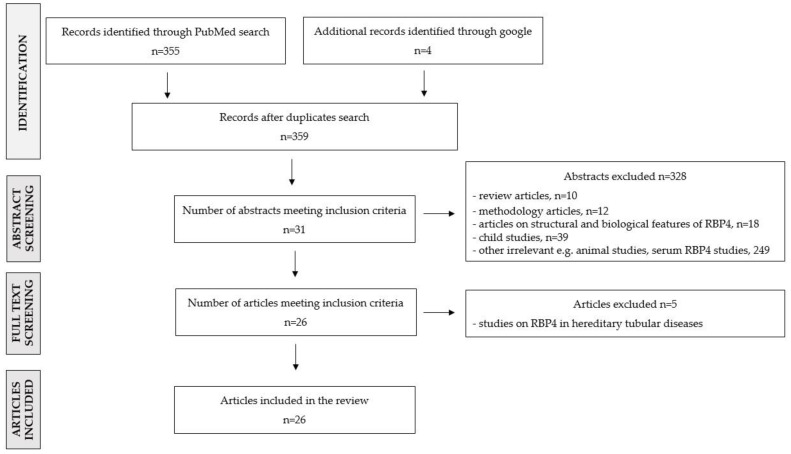
Flowchart of the literature selection process.

In order to extract the representative scientific literature concerning uRBP4 levels in humans in disease states, we searched the electronic bibliographic PubMed database (on 31 March 2022) using the following search terms: ((((urine RBP4) OR (urinary rbp4)) OR (uRBP4)) OR (urine retinol binding protein 4)) OR (urinary retinol binding protein 4), and filter options: full-text article (original article); species: human; language: English; article type: classical article. The initial search yielded 355 articles published between 1972 and 2022. Research papers were sorted by publication date, extracted (title + abstract), and manually verified. Additionally, a google search was performed to identify relevant articles not found in PubMed (*n* = 4). Pediatric/adolescent studies (age < 20 years) were excluded, as this review aimed to focus solely on the adult population in which urinary RBP4 levels are stabilized in a normal state and are not disturbed by maturation. Research that concentrated on uRBP4 response to therapy/toxic substance exposure (e.g., cadmium) was also not discussed in this work. In total, 26 original articles met all the eligibility criteria and were analyzed for the purpose of this review (all studies are summarized in Supplementary Table S2 and in the main text). Figure 1 depicts the flowchart outlining the article selection strategy.

## 3. Results

### 3.1. Urinary RBP4

#### 3.1.1. uRBP4 Levels in Health

In normal, voided urine, RBP4 presents at a relatively high abundance and constitutes nearly 8.5% of the urinary proteome [18]. RBP4 molecules can be detected in total urine, urine supernatant (debris-deprived urine), as well as in urinary extracellular vesicles (EVs, formerly called exosomes) [19,20,21]. There are three RBP4 isoforms encountered in human urine, namely full-length RBP4 and its two C-terminal truncated derivatives, RBP4-L (lacking Leu-183) and RBP4-LL (deprived of Leu-183 and Leu-182). RBP4 variants occur in normal urine in a 2:2:1 ratio and can be distinguished and quantified using mass-spectrometric analysis [22]. It has not yet been reported which RBP4 isoforms reside inside extracellular vesicles.

Urinary excretion of RBP4 is the most intense and variable in the infancy period between 0 and 6 months, which is due to renal tubule immaturity and reduced reabsorption efficiency (Supplementary Table S1) [23,24,25]. In newborns (up to 1 month), most of the glomeruli (80–96%) are immature, while in the later period, their number sharply decreases, reaching 2–59% in infants aged 2–6 months, and less than 10% in children up to 2 years [26]. In children above 2 years and adults, the concentration ranges of uRBP4 are similar and much more “narrow” compared with infants (Supplementary Table S1) [13,27,28]. Previous studies have shown that there are no gender-related differences in uRBP4 levels, either in adults or children, and that there is a low intraindividual, day-to-day variation in RBP4 excretion [28,29,30,31]. In adolescence (<20 years old), uRBP4 levels are often elevated and tend to normalize (decrease) after reaching puberty. Thus, uRBP4 interpretation in newborns, children, and adolescents is different from those in adults [32]. Possibly, the use of uRBP4 as a disease biomarker should be limited to adults (>20 years old) when the physiological levels of RBP4 have already stabilized. So far, no large multicenter trial has been launched with the purpose of determining the reference concentration ranges of urinary RBP4 in “healthy” adults. No convincing data exist on the possible confounding impact of variables such as ethnicity, diet, obesity, pregnancy, stimulants (caffeine, nicotine, alcohol), popular medications, etc. on uRBP4 levels in normal human urine.

#### 3.1.2. uRBP4 Levels in Diseases Involving the Kidneys

As the most sensitive indicator of proximal tubule “health”, uRBP4 bears a tremendous potential to become a universal screening biomarker of human diseases, in which renal tubule impairment plays a significant part. Urinary RBP4 levels directly reflect the magnitude of renal tubule malfunction and, thus, may provide useful information regarding the severity of the disease and the dynamic of its progression or remission. RBP4 presents in urine at extremely high concentrations and undergoes the most intense extraction (>10^4^-fold above “normal range”) when the proximal tubules fail to reabsorb low-molecular-weight proteins (e.g., heavy tubular proteinuria in renal Fanconi syndrome, FS). Urinary RBP4 expression pattern reflects the reabsorption capacity of renal tubules only if the glomerular filtration rate is normal or slightly impaired (i.e., serum creatinine <2 mg/dL, normo- and microalbuminuria, eGFR ≥ 60 mL/min/1.73 m^2^) [12]. Overextraction of protein may also occur under preserved proximal tubule function, i.e., when the reabsorptive capacity of the proximal tubules is exceeded (such as in cast nephropathy) or in severe proteinuria (e.g., in renal glomerular failure) [12,22,33]. Fluctuations in the number of plasma RBP4 seem to exert little or no impact on the amount of RBP4 in the urine [14]. Pathological states accompanied by tubular dysfunction are numerous, extremely heterogeneous, and predominantly acquired, such as some types of primary glomerular diseases, acute kidney injury (AKI), renal cell carcinoma (RCC), type 2 diabetes mellitus (T2DM), acquired Fanconi syndrome, and many others. Hereditary tubular diseases are rare and comprise a group of over 50 pathologies (e.g., cystinosis, Dent disease, Lowe syndrome, autosomal dominant FS) caused by genetic variants disrupting the function of renal tubular transporter genes [34]. Due to the rareness of inherited tubulopathies, as well as the limited body of published literature concerning the role of urinary RBP4 in congenital tubular disorders, this aspect will not be further discussed.

Below we provide an overview of the most common acquired disease states in which urinary RBP4 measurements were found to facilitate disease detection, risk stratification, and/or disease monitoring.

#### 3.1.3. Kidney Diseases

##### Primary Glomerular Diseases

Glomerular diseases (glomerulopathies) (GDs) are a heterogeneous group of disorders characterized by pathological alterations in glomerular structure and function. GDs may be diagnosed as primary if they are limited solely to the kidney, e.g., IgA nephropathy (IgAN), focal segmental glomerulosclerosis (FSGS), minimal change disease (MCD), membranous nephropathy (MN), and membranous proliferative glomerulonephritis (MPGN), or as secondary if they are a manifestation of systemic disease, such as diabetes, hypertension, amyloidosis, vasculitis, or lupus (e.g., diabetic kidney disease, secondary FSGS, secondary IgAN, lupus nephropathy) [35]. Glomerulopathies are the commonest cause of chronic kidney disease (CKD) and, in consequence, end-stage renal failure (end-stage renal disease, ESRD) worldwide. Renal biopsy is the “gold standard” for GDs diagnosis and subsequent management. Unfortunately, glomerular disease classification using these approaches still remains greatly heterogeneous in terms of severity, clinical course, response to treatment, and clinical outcome [36]. Novel, effective (preferably noninvasive) biomarkers are critically needed to aid early diagnosis, prognosis, and prediction in glomerular diseases. Because tubular injury may contribute to the loss of renal function in GDs, urinary tubular markers (including RBP4) seem to constitute a promising source of biomarkers for glomerular diseases [36].

Mastroianni-Kirsztajn et al. revealed, for the first time, that uRBP4 levels are significantly raised in certain types of GDs, i.e., FSGS, MN, MPGN, and IgAN. In the above-mentioned diseases, uRBP4 was able to predict loss of renal function efficiently and independently, especially at 6 months of follow-up, when the patients were pharmacologically stabilized. Conversely, in MCD, mesangial proliferative glomerulonephritis, poststreptococcal glomerulonephritis (PSGN), and glomerular hematuria, the expression patterns of uRBP4 overlapped with those of healthy controls, suggesting the well-preserved function of the renal tubules is these conditions [37]. Similar to Mastroianni-Kirsztajn et al., our group also observed significantly higher levels of uRBP4 in FSGS and IgAN subjects in relation to healthy controls. The protein was significantly more abundant in FSGS than in IgAN patients. Interestingly, uRBP4 concentration ranges in IgAN overlapped with those of disease controls, i.e., renal cell carcinomas (clear cell renal cell carcinoma, ccRCC; chromophobe renal cell carcinoma, chRCC) (Supplementary Table S2) [21]. Kalantari et al. employed two different quantitative proteomic strategies to characterize the urinary proteome of early-stage IgAN (class II–IV according to the Haas and Lee classification) and advanced IgAN (class V). The authors developed a signature composed of uRBP4 and 10 other proteins that could adequately predict IgAN severity (80% sensitivity and 100% specificity in comparison with the Oxford classification). Urinary RBP4 was highly overrepresented in the advanced IgAN and comprised the most differentially regulated component of the signature (Supplementary Table S2) [38]. Zhang et al. observed higher uRBP4 levels in FSGS with nephrotic syndrome patients compared with MCD subjects with similar levels of proteinuria (Supplementary Table S2). In the FSGS group, the amount of RBP4 was positively correlated with serum creatinine concentration, the extent of proteinuria, and the degree of acute tubulointerstitial lesion. Interestingly, FSGS subjects with higher concentrations of uRBP4 presented worse treatment outcomes than those with lower amounts of the protein (Supplementary Table S2) [39]. Recently, urinary proteomic profiling performed by Araumi revealed that the combination of uRBP4 and urinary SH3 domain-binding glutamic acid-rich-like protein 3 (uSH3BGRL3) could clearly distinguish between MCD with nephrotic syndrome and diabetic kidney disease (DKD) (AUC = 0.974). The classifier was found to outperform the clinical selectivity index (SI), which is based on serotransferrin and immunoglobulin G [40].

Undoubtedly, uRBP4 represents potential as a diagnostic, prognostic, and/or predictive tool in glomerular diseases. However, further research is necessary to collect enough evidence for its clinical applicability.

#### 3.1.4. Acute Kidney Injury

Acute kidney injury (AKI) is a highly lethal (~50% of AKI cases) syndrome characterized by a sudden decrease in kidney function. AKI is usually caused by acute tubular necrosis (ATN) or prerenal azotemia (PRA). ATN involves damage to the renal tubules and is usually initiated by ischemia or nephrotoxic agents (recently, it was also frequently observed in COVID-19 patients). PRA is caused by a decrease in renal blood flow (hypoperfusion) and is characterized by abnormally raised levels of nitrogen waste products in the blood (e.g., urea, creatinine). PRA is frequently diagnosed in patients with heart attack, severe burns, liver failure, and severe dehydration [41,42]. Early and accurate detection of AKI, and its direct cause, is crucial to ensure prompt, appropriate management. Unfortunately, the clinical gold standard for the diagnosis of AKI, i.e., serum creatinine concentration measurement, lacks sufficient sensitivity in its earliest stages, and there are no other markers available enabling early detection of AKI. The search for such indicators is heavily hampered by the heterogeneity of AKI patients in relation to comorbidities, underlying etiology, age, prior treatment, etc. [41,43].

Varghese et al. applied two-dimensional electrophoresis (2-DE) to identify urinary protein/-s indicative of the two main causes of AKI (ATN vs. PRA). The authors found that an accurate diagnosis might be made by interpreting uRBP4 and urinary albumin using a complex algorithm (AUC = 0.88) [41]. Unfortunately, this finding has never been validated or commented on by other research groups. In another study, quantitative proteomic analysis revealed that uRBP4 concentration is significantly raised in stage 1 (T1) AKI and progressively drops toward normal levels during remission. It was also noted that RBP4 responds quicker to recovery than serum creatinine concentration [43]. Unfortunately, similar to the previous case, the data collected by Gonzalez-Calero has never been subjected to independent validation.

Certainly, more research is needed to ultimately comprehend the role of uRBP4 in AKI and to assess RBP4 usefulness in the early diagnosis of AKI and recovery prediction.

#### 3.1.5. Renal Allograft Dysfunction

Kidney transplantation (KT) is a treatment of choice in patients with ESRD and is associated with lower mortality and better quality of life compared to dialysis. Unfortunately, a significant number of transplant recipients experience renal allograft failure at various time points (~20% within 5 years and ~50% within 10 years), and there are no robust biomarkers able to predict allograft survival [44]. Early detection/prediction of the graft dysfunction is a prerequisite to initiating adequate preventive therapy and avoiding biopsy. In 2003, Hosaka et al. showed that uRBP4 might facilitate the identification of renal transplant patients at higher risk of developing graft dysfunction in the first year postsurgery. Patients with normal graft function and a uRBP level of >0.6 mg/L during the first 3 months after transplantation were more likely to experience graft dysfunction (Supplementary Table S2) [45]. A year later, the group of Câmara published the results of a 5-year prospective study in which uRBP4 expression was monitored in renal transplant patients with stable graft function at the time of enrollment. Similar to Hosaka, the authors found that uRBP4 was useful in predicting long-term graft survival. Patients with a uRBP4 level of >0.4 mg/L had a five times greater risk of developing chronic graft nephropathy (CAN, a leading cause of allograft loss) and graft loss within 5 years postsurgery than those with lower levels of uRBP4. Interestingly, the increase in uRBP4 concentration preceded the development of CAN by almost 2 years, giving the chance to initiate “preventive treatment” (Supplementary Table S2) [46]. In another study, higher levels of uRBP4 (≥2.85 mg/L) were predictive of worse allograft survival in patients with transplant glomerulopathy (TG, posttransplant, rare pathological lesion associated with poor survival). However, in this particular case, uRBP4 did not outperform the estimated glomerular filtration rate (eGFR), which appeared to be the most powerful predictor of allograft survival in TG (Supplementary Table S2) [47]. In 2021, Jeon et al. reported that uRBP4 levels are tightly associated with renal function in kidney recipients and that the protein can be used to predict rapid renal function decline. By employing the SWATH-MS approach (discovery phase) and ELISA (validation phase), the authors showed that the concentration range of uRBP4 is strikingly different between subjects with allograft dysfunction and transplant patients with normal renal function. The urinary RBP4 to creatinine ratio was found to be inversely and significantly associated with the condition of the allograft (r = −0.54, *p* < 0.001; odds ratio 7.59, confidence interval 2.04–36.7, *p* = 0.005). When adjusted with recipient age, sex, donor age, number of HLA mismatch, and acute rejection episode, uRBP4 appeared to be a significant risk factor for rapid renal function decline (odds ratio 9.43, confidence interval 1.99–65.65, *p* = 0.01) (Supplementary Table S2) [48].

The number of studies examining the usefulness of uRBP4 in the prediction of long-term postrenal transplantation complications is scarce. Much more research is needed to validate the reported findings and to develop standardized procedures for measuring uRBP4 in kidney transplant recipients.

#### 3.1.6. Chronic Kidney Disease

Chronic kidney disease (CKD) is a heterogeneous group of disorders whose common feature is the gradual loss of kidney function over a period of months or years. CDK impacts approximately 8–16 (%) of the population worldwide and is currently understood as a serious public health problem [49]. The definition of CDK is based on the presence of urinary abnormalities (proteinuria, erythrocytes), decreased kidney function (reflected by eGFR decline), electrolyte imbalance, or kidney abnormalities revealed by imaging techniques persisting for at least 3 months [50,51]. CKD is classified on the basis of GFR level into five stages, ranging from stage 1–3 (early stage, usually asymptotic), stage 4 associated with severely reduced kidney function, to stage 5 (GFR < 15 mL/min/1.73 m^2^), also called end-stage renal disease (ESRD). The predominant causes of CDK are: glomerulopathies, diabetes mellitus, and chronic hypertension [51,52]. The current approaches for CKD diagnosis and monitoring are inefficient in the early detection of CKD and predicting which patient will progress toward ESRD [53]. Domingos et al. were the first to evaluate uRBP4 levels in a large, heterogeneous CKD population, encompassing stage 3 and stage 4 CKD of varying etiology (excluding glomerulonephritis and transplantation). The group found that RBP4 is significantly associated with renal function in CKD in general and may serve as an independent predictor of CKD progression [33]. Recently, the group run by Fernando measured uRBP4 and seven other chosen renal urinary proteins in CKD of known and uncertain etiology and found that RBP4, alpha 1 microglobulin (A1M), and kidney injury molecule-1 (KIM1) represent a minimum marker combination for differentiating all CKD categories from healthy controls. Importantly and interestingly, another biomarker panel consisting of uRBP4, osteopontin (OPN), and KIM1 was found to have great performance for distinguishing patients with CKDu from other CKD categories. The classifier was superior to any other existing noninvasive indicator [54].

A large validation study, including all possible CKD categories and reference groups is required to assess whether uRBP4 testing could serve as a useful screening tool for CKD.

#### 3.1.7. Renal Cell Carcinoma

Renal cell carcinoma is the most common and most lethal group of urological malignancies arising from the renal tubular epithelial cells [55]. The predominant subtypes of RCC are clear cell renal cell carcinoma (ccRCC), papillary RCC (pRCC), and chromophobe renal cell carcinoma (chRCC). In most cases, RCCs are asymptomatic in the earliest stages and diagnosed incidentally during imaging investigations [56]. Unfortunately, around 25% of RCC patients possess metastatic disease already at the time of diagnosis, and approximately 30% of subjects undergoing surgery for early-stage disease develop metastases within a year [57,58]. Five-year survival rates for metastatic RCC are poor and range from 0 (untreated metastatic RCC) to 20% (treated metastatic RCC) [59]. If detected early, RCC is sufficiently treatable, and the risk of disease recurrence is minor. Unfortunately, there is a lack of sensitive, noninvasive biomarkers for early diagnosis, differential diagnosis, and prognosis of RCC. Because renal cell carcinomas originate from renal tubules, the tubular markers seem to comprise an important source of putative clinical indicators for RCC [60].

To our knowledge, there are only two studies evaluating uRBP4 expression status in renal cancer patients. Recently, our group analyzed uRBP4 expression patterns in RCC subjects (ccRCC and chRCC) and compared them with uRBP4 levels in healthy controls and men with prostate cancer (disease control group). Our data demonstrated that uRBP4 levels are significantly increased in RCC and that RCC diagnosis cannot be stated based on uRBP4 alone due to overlapping expression patterns between RCC and reference groups [21]. Because some RCC patients presented “normal” levels of uRBP4, we hypothesize that tubular injury may not be an obvious feature of RCC tumors. Santorelli et al. mined the urinary proteome of ccRCC patients with lesions of varying severity (low-grade vs. high-grade tumors, early-stage vs. advanced-stage RCC) and found that uRBP4 levels correlate positively with ccRCC stage but not ccRCC grade. RBP was upregulated almost three-fold in advanced tumors and comprised one of 79 proteins “associated” with tumor progression [61].

Undoubtedly, additional investigations are necessary to assess whether uRBP4 testing could improve the detection rate of nonadvanced RCC and disease management.

#### 3.1.8. Systemic Diseases Affecting Kidneys

##### Type 2 Diabetes Mellitus

Type 2 diabetes mellitus is the predominant form of diabetes (~90% of all cases) related to insulin resistance, which is usually diagnosed in older adults. T2DM is a progressive disorder associated with a number of micro- and macrovascular complications. The most common, long-term microvascular consequence of T2DM (diagnosed in ~50% of patients) includes diabetic kidney disease (DKD, formerly diabetic nephropathy, DN), which tends to progress to CKD and eventually to ESRD (in around 30–45% of DKD patients) [13,62,63]. The “gold standard” for the early diagnosis of DKD is microalbuminuria, which is, however, not ideal due to limited diagnostic accuracy. A significant number of T2DM patients with impaired renal function do not present preceding albuminuria (30–40%), and, inversely, those with elevated albumin do not always develop renal disease [64,65]. Sensitive biomarkers of DKD that outperform albuminuria are needed to provide earlier diagnosis and a more accurate prognosis in diabetic patients.

It is well recognized that tubular injury plays a significant part in the pathophysiology of DKD. Yaqoob et al. were the first to show that renal tubular damage precedes microalbuminuria and comprises a critical step in DKD initiation. The authors observed significantly higher levels of low-molecular-weight proteins (including RBP4) in the urine of normoalbuminuric T2DM patients with endothelial dysfunction compared with T2DM subjects with normal endothelial function and healthy controls (Supplementary Table S2) [13]. Similar observations were made by Park, revealing that uRBP4 concentrations are raised at the prediabetic stage and remain correlated with insulin resistance, inflammation, and microalbuminuria (Supplementary Table S2) [66]. In recent decades, a vast number of studies provided supporting evidence that uRBP4 comprises a much more powerful indicator of early-stage DKD than microalbuminuria and that it can effectively predict DKD progression and patient outcomes (Supplementary Table S2) [31,65,67,68,69,70]. A large, well-designed clinical validation study is required to ultimately assess the diagnostic and clinical utility of uRBP4 measurements in type 2 diabetic patients.

#### 3.1.9. Obesity

Obesity, defined as an abnormal or excessive fat accumulation in the body, remains the main risk factor for diabetes, hypertension, cardiovascular complications, and renal cancer. According to the World Health Organization (WHO), in 2016, approximately 650 million adults suffered from obesity worldwide (www.who.int (accessed on 22 May 2022)). Not every obese individual will develop obesity-related complications (~30% of cases), and great efforts are being made to develop biomarkers identifying the “high-risk” subpopulation [71]. Recently, a quantitative comparative analysis of the urinary proteomes between metabolically healthy obese subjects (MHO) and metabolically unhealthy obese patients (MUHO, higher insulin level and insulin resistance) revealed that uRBP4 concentration is slightly increased in the MUHO group (fold change 1.5, *p* < 0.05). The concentration of uRBP4 was found to correlate with RBP4 elevation in the serum, as well as altered insulin sensitivity in the unhealthy subjects (Supplementary Table S2) [72]. The study by Benabdelkamel was the first and the only one showing a possible link between the level of uRBP4 and the complications related to obesity. Because the study included a small number of subjects, and RBP4 had the smallest fold change among the 54 differentially expressed proteins, further research is necessary to clarify whether uRBP4 measurements may provide any benefit in predicting the risk of obesity-related complications. It also remains to be explored whether uRBP4 measurements performed for other medical indications may be confounded by obesity.

#### 3.1.10. Preeclampsia

Preeclampsia (PE) is a serious complication affecting 3–10 (%) of all pregnancies, characterized by newly diagnosed hypertension after 20 weeks of gestation and one of the following complications: proteinuria, liver affection, and nervous system involvement. The molecular mechanism behind PE is an imbalance in angiogenic and antiangiogenic factors, leading to systemic endothelial dysfunction. PE frequently affects the kidneys by causing damage to the glomeruli, renal proximal tubules, and vascular endothelium [73,74,75]. The disease strongly predisposes to CKD and increases the risk of ESRD postpregnancy by four-fold [75]. There is a lack of noninvasive markers allowing the forecast of renal complications during and after PE. The concentration of uRBP increases during uncomplicated pregnancy and reaches the highest levels in the third trimester. In some cases, the amounts of uRBP4 observed in the last trimester are similar to those recorded in pathological conditions (e.g., in nonpregnant diabetic patients) [76]. In 2012, Facca et al. were the first to evaluate uRBP4 levels in pregnant women with PE (third trimester), finding its abnormally high amounts (Supplementary Table S2) [77]. A year later, the group run by Xiao confirmed this finding and additionally revealed that the combination of uRBP4, serum cystatin C, urine NGAL, and urine KIM-1 detects PE-related renal injury with 100% sensitivity and 98.2% specificity [78]. Further research is necessary to comprehend the dynamics of changes in the concentration of uRBP4 in the course of PE and after pregnancy. There is a necessity to evaluate the performance of uRBP4 in predicting renal diseases in women with a history of PE and also to verify if the pregnancy may interfere with uRBP4 measurements performed for other medical reasons.

#### 3.1.11. Coronavirus Disease 2019

Coronavirus disease 2019 (COVID-19), caused by a novel severe acute respiratory syndrome coronavirus-2 (SARS-CoV-2 virus), is an unpredictable illness that may be asymptomatic as well as fatal. COVID-19 first occurred in Wuhan, China, in November 2019 and spread globally, causing a pandemic. According to WHO registries, up to 5 May 2022, there were over 500 million COVID-19 cases and more than 6 million COVID-19-related deaths recorded worldwide [79]. SARS-CoV-2 is a highly transmissible virus that primarily affects the upper and the lower respiratory tract (particularly the lungs) but may damage virtually every part of the body. The pathogen invades human cells via the angiotensin-converting enzyme (ACE2) receptor, which is ubiquitously expressed throughout the body, including the kidneys [80]. Over 70% of patients hospitalized due to a severe form of COVID-19 present abnormal urine parameters (proteinuria, hematuria, and/or leukocyturia), and as much as ~50% of them develop AKI and, subsequently, ~19% require dialysis [81,82]. There is a great need for clinical tools that could predict early AKI during COVID-19 in order to improve patient outcomes. Because SARS-CoV-2 initiates AKI by invading and injuring renal tubular epithelial cells, the tubular markers (including uRBP4) comprise a potential source of AKI predictors [83,84]. Karras et al. were the first and the only ones to evaluate the performance of uRBP4 for detecting early AKI in hospitalized COVID-19 patients. The group found that higher RBP4 levels (≥0.03 mg/mmol) at the time of hospital admission had a strong positive correlation with the incidence of AKI, admission to the intensive care unit, and the risk of death (Supplementary Table S2) [82]. Further research is needed to confirm this original finding, as well as to verify whether routine uRBP4 assessment after a positive COVID-19 test could facilitate the identification of subjects at risk of renal complications.

## 4. Conclusions

The discovery of RBP4 in human urine by Kanai in the late 1960s initiated “an avalanche” of research into it in the context of its characteristics, as well as its applicability as a renal disease biomarker. A PubMed search using the keywords urine RBP4, urinary RBP4, urine retinol binding protein 4, urinary retinol binding protein 4, and uRBP4 yielded a list of 355 full-text English articles on 31 March 2022. Previous research into uRBP4 has consistently shown that the protein is the most sensitive functional marker of proximal renal tubules, which can detect even minor renal tubular impairment. Numerous studies provided strong evidence that uRBP4 can facilitate the early diagnosis, differential diagnosis, and/or prognosis of a wide variety of renal diseases accompanied by renal tubular dysfunction, e.g., some types of glomerular diseases, AKI, renal graft dysfunction, chronic kidney disease, and renal cell carcinoma. Unfortunately, these encouraging findings have not yet been sufficiently validated and translated into clinical practice. The huge heterogeneity across studies in terms of research design (urine collection and processing protocols, analytical assays employed, etc.), population composition, and data analysis (differences in statistical methods, data normalization strategies, concentration units, and concentration cutoffs) makes it impossible to directly compare the results and draw definitive conclusions about the clinical validity and usefulness of uRBP4. There is a need to develop and implement well-standardized protocols to ensure reproducibility, comparability, and generalizability across studies. Hopefully, future well-planned validation studies will decipher the true clinical value of uRBP4.

## Data Availability

Not applicable.

## Supplementary Materials

The following supporting information can be downloaded at: https://www.mdpi.com/article/10.3390/ijerph19169878/s1. Table S1: urinary RBP4 concentrations according to age group, Table S2: summary of the most important studies examining the association between uRBP4 levels and human renal diseases.
